# Construct Validation of the Rainbow Model of Integrated Care Measurement Tool in Dutch Primary Care for Older Adults

**DOI:** 10.5334/ijic.6739

**Published:** 2023-02-15

**Authors:** Dorien L. Oostra, Minke S. Nieuwboer, Jeroen H. M. Janssen, Marcel G. M. Olde Rikkert, Pim P. Valentijn, Marieke Perry

**Affiliations:** 1 Radboud university medical center, Radboud Institute for Health Sciences, Department of Geriatric Medicine, Nijmegen, The Netherlands; 2 Radboudumc Alzheimer Center, Radboud university medical center, Nijmegen, The Netherlands; 3 HAN University of Applied Sciences, Academy of Health and Vitality, Nijmegen, The Netherlands; 4 Radboud university medical center, Donders Institute for Brain Cognition and Behaviour, Department of Geriatric Medicine, Nijmegen, The Netherlands; 5 Department of Health Services Research, Care and Public Health Research Institute (CAPHRI), Faculty of Health, Medicine and Life Sciences, Maastricht University, Maastricht, The Netherlands; 6 Essenburgh Research & Consultancy, Essenburgh Group, Harderwijk, The Netherlands; 7 Radboud university medical center, Donders Institute for Brain Cognition and Behaviour, Department of Primary and Community Care, Nijmegen, The Netherlands

**Keywords:** integrated care, measurement tool, elderly care, primary care, care networks, interprofessional collaboration

## Abstract

**Introduction:**

Care integration in primary elderly care is suboptimal. Validated instruments are needed to enable the implementation of integrated primary care. We aimed to assess construct validity of the Rainbow Model of Integrated Care measurement tool (RMIC-MT) for healthcare professionals working in an integrated primary elderly care setting in the Netherlands.

**Methods:**

In a cross-sectional study, the RMIC-MT, a 36-item questionnaire covering all domains of the Rainbow Model of Integrated Care (RMIC), was sent out to local networks of primary elderly care professionals. Confirmatory factor analysis with maximum likelihood estimation was used for the validation of the factor structure of the RMIC-MT. Model fit was assessed by the chi-square test and fit indices.

**Results:**

The RMIC-MT was completed by 323 professionals, primarily general practitioners, community nurses, practice nurses, and case managers. Confirmatory factor analysis and corresponding fit indices showed moderate to good fit, thereby confirming a nine factor model with a total of 36 items.

**Conclusions:**

The RMIC-MT is promising for the primary elderly care setting in the Netherlands. It can be used for evaluating integrated care initiatives in a primary care setting, thereby contributing to implementation of integrated primary elderly care.

## Introduction

The number of older persons living at home with complex healthcare needs is rising. Currently, care for older persons is fragmented as a result of suboptimal communication and coordination among the multiple healthcare professionals that are often involved in a primary care setting [1,2,3]. It is considered essential to cross the disciplinary boundaries between professionals to enable care coordination between multiple healthcare services [2]. Integrated care is an important strategy to overcome fragmentation of care. A framework that captures the concept of integrated care and describes its key domains is the Rainbow Model of Integrated Care (RMIC) [4] that was specifically developed for evaluating integrated primary care settings. Improving care integration and thereby quality of care could contribute to a more sustainable, patient-friendly, and affordable health care system [5,6].

To enable implementation of integrated care for older people it is essential to measure integrated care performance in practice [7,8,9]. Nevertheless, validated measurement instruments are lacking [10,11,12,13,14], because of the complexity of integrated care as a construct [16]. Available instruments are thus either of poor psychometric quality [11,14] or are too elaborate for use in practice [15]. Based on the RMIC framework, the Rainbow Model of Integrated Care Measurement Tool (RMIC-MT) was developed [16]. This questionnaire was tested for internal consistency and construct validity internationally for renal care and in Chinese primary care [16,17]. Since the RMIC-MT is a valid measure to evaluate integrated care in several care settings, it may have the potential to validly measure the integration of elderly care networks and support professionals to improve their interprofessional collaboration and eventually the quality of care for older persons living at home. Therefore, we aim to assess the construct validity of the RMIC-MT for healthcare professionals working in an integrated primary elderly care setting in the Netherlands.

## Methods

### Study design

A cross-sectional study design was used for construct validation of the RMIC-MT for healthcare professionals in elderly care. Data were collected between February 2020 and February 2021.

### Setting and study population

Participants were healthcare professionals active in primary elderly care networks throughout the Netherlands. Networks were recruited via two routes: 1) via the DementiaNet study and 2) via newsletters of general practitioner- and geriatric specialists organisations and all regional elderly care networks in the Netherlands. The DementiaNet program [18] supports network development in primary elderly and dementia care. These networks include medical, care, and social professionals and provide interprofessional care to a shared caseload of persons with dementia. More information about the DementiaNet program is available elsewhere [18,19]. An overview of the networks’ geographical location can be found in Appendix I.

We used convenience sampling with the following inclusion criteria for the primary elderly care networks: 1) at least a general practitioner practice and community nurse present, 2) professionals from at least two organisations present, 3) a network leader is appointed, 4) all network members share a caseload of at least two persons, 5) work agreements are made or a regular multidisciplinary meeting is held, and 6) at least three persons of the network are willing to complete the questionnaire. All networks meeting the inclusion criteria were invited to participate, independent of their development. When inclusion criteria were met, network members or the contact person were invited via email to participate in the study. All participating networks received a feedback report, enabling them to identify areas of improvement.

### Sample size

To determine the a priori sample size we used the rule of thumb of including 10 subjects per questionnaire item [20]. Given that the RMIC-MT for professionals has 36 items our targeted sample size was 360 participants.

### Theoretical framework

The RMIC is a validated framework that was developed to operationalise the construct of integrated primary care. Primary care is considered the focal point for integrating various medical, care, and welfare services close to people’s homes. The RMIC defines the necessary integration mechanisms by also emphasizing its final (Triple aim) outcomes [4]. Different integration domains are specified in the RMIC, making it possible to identify specific areas for improvement. The RMIC describes three categories of integrated care: the scope, type, and enablers of integration, consisting of eight domains. The scope entails the person-focused and population-focused view of health issues at the micro and meso-macro level respectively. The type of integration refers to four domains: clinical integration, professional integration, organisational integration, and system integration. Enablers entails functional and normative integration. Further explanation of the RMIC can be found in Appendix II.

### RMIC Measurement Tool

The RMIC-MT consists of a 36-item questionnaire for professionals [21], covering all aspects of integrated care as described by the RMIC [4]. The development and international validation of the RMIC-MT is described elsewhere [16]. The RMIC-MT was translated to Dutch using forward translation by an independent translator and adapted to the Dutch primary care setting for older adults. To optimize applicability in primary elderly care the Dutch RMIC-MT was reviewed and pilot-tested by six experts in the field of primary elderly care, including three authors (DO, MP, and MN) and three external reviewers with a healthcare professional (general practitioner, community nurse, and case manager) and managerial backgrounds. The version of the RMIC-MT used in this study can be found elsewhere.

The RMIC-MT has the following domains: Person-centeredness (e.g. needs assessment), population-centeredness (e.g. population screening), clinical coordination (e.g. personal care plan), professional coordination (e.g. multidisciplinary team), organisational coordination (e.g. inter-organisational partnerships), system coordination (e.g. policy and financing), technical competence (e.g. interoperable medical records), triple aim (e.g. outcome assessment), and cultural competence (e.g. collaboration culture). We reformulated the name of the domain ‘triple aim’ to ‘results-oriented’, which better describes the items of this domain. The RMIC-MT consisted of 36 items divided over these nine integration domains (see Appendix III for the number of items per domain). The RMIC-MT assessed how formal providers of primary care for older adults perceived their network’s ability to deliver integrated care on a five-point Likert scale (options were: don’t agree at all, don’t agree, neutral, agree, fully agree; I don’t know or never, rarely, sometimes, often, always). In this study, we added the ‘I don’t know’ option. At the end of the RMIC-MT questionnaire information on professional background was collected.

### Data collection

The RMIC-MT questionnaire was sent by email between February 2020 and February 2021. The RMIC-MT questionnaires were completed online using a web-based survey platform. Consent was asked at the beginning of the questionnaire. To prevent missing answers all questions were mandatory. The response rate per network was assessed through network-specific codes assigned to each questionnaire. Participants received a reminder after one week and after two weeks when the questionnaire was not completed.

### Analysis

Number and percentage were calculated for the background characteristics of the participants. For the final dataset we merged the ‘I don’t know’ scores with the ‘neutral’ option, as the previous studies did not have an ‘I don’t know’ option and both options express the lack of a directed opinion. We first performed an Exploratory factor analysis (EFA) to explore the underlying factor structure. Confirmatory factor analysis (CFA) was used to build upon the previous results of the RMIC-MT [16] that consisted of 36 items and nine factors [16,17].

Maximum likelihood estimation was used on the variance-covariance matrix and model fit was assessed using the chi-square statistic and goodness-of-fit indices. The fit indices that we used were the Comparative Fit Index (CFI) and Tucker Lewis Index (TLI) (both considered adequate if above 0.9 and good if above 0.95), Root Mean Square Error of Approximation (RMSEA, good if close to 0.06 or below), and Standardized Root Mean Square Residual (SRMR, good if close to 0.08 or below) [22]. RMSEA is an absolute fit index that assesses the difference between the hypothesized and a perfect model [23]. CFI and TLI are incremental fit indices which compare the fit of the data and the hypothesized model [23]. The SRMR is the average of the standardized residuals between the covariance matrix of the data and the model [24]. These fit indices were considered in combination, as good model fit entails meeting all these criteria [22,25]. Significance of the chi-quare statistic indicates good model fit when the p-value is not significant (i.e. above .05). R Studio version 3.6.2 [26] with the Lavaan package [27] were used for analysis.

### Ethical approval

The study was conducted according to the principles of the Declaration of Helsinki (2013). The research ethics committee of the Radboud university medical center stated that the study did not fall within the remit of the Medical Research Involving Human Subjects Act (WMO) (file number: 2019-5599).

## Results

### Participant characteristics

Three hundred twenty-three healthcare professionals started the survey, of which 282 completed the RMIC-MT. In total, 262 of 450 DementiaNet professionals participated (58% response rate), divided over 34 networks. Furthermore, 61 external professionals from 10 different networks participated. The majority of professionals were case managers (18%), community nurses (18%), general practitioners (12%), and practice nurses (11%). Median time needed for completing the RMIC-MT was 15 minutes (Inter Quartile Range = 13 minutes). Table 1 provides an overview of the participant characteristics.

**Table 1:** Characteristics of the primary healthcare professionals participating in the RMIC-MT for elderly care.


	PARTICIPANTS, N = 323

**Professional background, n (%)**	

General practitioner	40 (12%)

Community nurse	57 (18%)

Practice nurse	35 (11%)

Physiotherapist	9 (3%)

Geriatric specialist	12 (4%)

Occupational therapist	11 (3%)

Welfare worker	23 (7%)

Pharmacist	3 (1%)

Case manager	57 (18%)

Other	30 (9%)

Unknown	46 (14%)

**Number of networks, n (%)**	

DementiaNet	34 (77%)

External networks	10 (23%)

**Participants per network type, n (%)**	

DementiaNet	262 (81%)

External networks	61 (19%)

### Missing value pattern

Forty-one participants partly completed the RMIC-MT. Since all questions were set as required (see details in method section) these incomplete RMIC-MTs are all missing the answers to the later items. See Table 2 for an overview of the total number of responses per item. We used all the answers (as mentioned in Table 2) for the analysis, also from incomplete questionnaires.

**Table 2:** Summary measures of the 36 items of the RMIC-MT for elderly care.


ITEM	N	MEDIAN	MEAN	MODE	STANDARD DEVIATION	SKEWNESS	KURTOSIS

**Person-centeredness**							

Interpersonal trust	323	5	4.65	5	0.532	–1.197	0.427

Listening	323	4	4.45	5	0.568	–0.423	–0.785

Social circumstances	323	5	4.43	5	0.652	–0.842	0.239

Preference integration	323	4	4.02	4	0.696	–0.136	–0.588

Questioning	323	4	4.14	4	0.707	–0.364	–0.399

**Community centeredness**							

Community partnerships	318	4	4.38	4	0.612	–0.519	–0.170

Health promotion	318	4	3.93	4	0.882	–0.559	0.053

Community collaboration	318	4	3.75	4	0.856	–0.244	–0.419

Population needs	318	4	3.65	4	0.853	–0.207	–0.254

**Clinical coordination**							

Case management	307	4	3.73	4	0.964	–0.734	0.486

Follow-up of care	307	4	3.97	4	0.958	–0.862	0.489

Shared decision-making	307	4	4.30	5	0.814	–1.011	0.746

**Professional coordination**							

Interdisciplinary communication	299	3	2.73	3	0.698	0.187	0.119

Interdisciplinary fragmentation	299	3	2.65	3	0.645	–0.117	–0.118

Interdisciplinary coordination	299	3	2.75	3	0.662	0.193	–0.130

Interdisciplinary follow-up	299	3	2.85	3	0.580	–0.082	0.068

Interdisciplinary teamwork	299	3	2.77	3	0.670	0.311	0.428

**Organisational coordination**							

Inter-organisational coordination	298	3	3.26	3	0.785	–0.407	0.408

Inter-organisational resources	298	4	3.39	4	0.979	–0.490	–0.197

Inter-organisational staff	298	3	3.41	4	0.860	–0.632	0.557

**System coordination**							

Inter-organisational incentives	295	3	3.03	3	0.751	–0.153	0.374

Interdisicplinary incentives	295	3	3.07	3	0.750	–0.069	–0.047

Care coordination incentives	295	3	3.10	3	0.695	–0.257	0.572

**Results-oriented**							

Needs assessment	292	4	3.79	4	0.747	–0.787	1.492

Experience assessment	292	3	3.47	4	0.972	–0.173	–0.528

Quality objectives	292	4	3.72	4	0.832	–0.508	0.347

Monitoring & follow-up	292	4	3.67	4	0.846	–0.445	0.325

Outcome assessment	292	3	2.99	3	0.945	–0.145	0.265

**Technical competence**							

Interoperable IT tools	288	3	2.99	3	1.095	–0.309	–0.680

Interoperable EHRs	288	3	2.64	3	0.995	–0.174	–0.752

Data integration	288	3	3.07	3	1.030	–0.294	–0.345

Outcome transparency	288	3	2.76	3	0.920	–0.282	–0.034

**Cultural competence**							

Fellowship	282	4	4.23	4	0.613	–0.641	2.334

Teamwork	282	4	3.76	4	0.754	–0.424	0.603

Respect	282	5	4.68	5	0.539	–1.430	1.115

Support	282	4	3.64	4	0.820	–0.307	0.409


### Confirmatory factor analysis

The EFA results showed a similar factor structure compared to the previous studies [16,17] (Appendix IV). Therefore, we continued with the CFA. Table 3 shows the factor loadings of the RMIC-MT and Appendix V provides the item correlation matrix. The chi-square test was significant, indicating bad model fit: χ^2^(558) = 945.08, p < .001. Using the previously mentioned cut-off values, both RMSEA (0.046) and SRMR (0.055) are considered good, while CFI (0.895) and TLI (0.882) are slightly under their cut-off and are therefore considered moderate. Summary measures including median, mean, and standard deviations for all items are depicted in Table 2.

**Table 3:** Factor loadings and standard errors of the confirmatory factor analysis of the 36-item RMIC-MT for elderly care.


ITEM	N	ESTIMATE	SE

**Person-centeredness**			

Interpersonal trust	323	1.000	

Listening	323	1.417	0.166

Social circumstances	323	1.550	0.185

Preference integration	323	1.807	0.226

Questioning	323	1.909	0.234

**Community centeredness**			

Community partnerships	318	1.000	

Health promotion	318	1.811	0.209

Community collaboration	318	1.667	0.197

Population needs	318	2.036	0.238

**Clinical coordination**			

Case management	307	1.000	

Follow-up of care	307	1.116	0.122

Shared decision-making	307	0.580	0.092

**Professional coordination**			

Interdisciplinary communication	299	1.000	

Interdisciplinary fragmentation	299	0.868	0.081

Interdisciplinary coordination	299	0.802	0.084

Interdisciplinary follow-up	299	0.680	0.075

Interdisciplinary teamwork	299	0.858	0.088

**Organisational coordination**			

Inter-organisational coordination	298	1.000	

Inter-organisational resources	298	1.361	0.177

Inter-organisational staff	298	1.233	0.159

**System coordination**			

Inter-organisational incentives	295	1.000	

Interdisicplinary incentives	295	1.041	0.062

Care coordination incentives	295	0.928	0.058

**Results-oriented**			

Needs assessment	292	1.000	

Experience assessment	292	1.188	0.120

Quality objectives	292	1.272	0.107

Monitoring & follow-up	292	1.238	0.107

Outcome assessment	292	1.027	0.116

**Technical competence**			

Interoperable IT tools	288	1.000	

Interoperable EHRs	288	1.021	0.098

Data integration	288	0.862	0.147

Outcome transparency	288	0.741	0.129

**Cultural competence**			

Fellowship	282	1.000	

Teamwork	282	1.446	0.186

Respect	282	0.524	0.096

Support	282	1.307	0.177


SE = Standard Error.

## Discussion

### Principal findings

This study provides the first assessment of the construct validation of the Dutch RMIC-MT version for professionals working in the integrated primary elderly care setting. In the CFA, the nine factors of the RMIC-MT passed the majority of goodness-to-fit tests. This makes the RMIC-MT’s utility promising, but not yet ready to assess integrated care in daily practice.

### Comparison with existing evidence

We were able to build upon previous validations of the RMIC-MT in different countries, languages, and for different conditions [16,17,28]. Although we were not able to successfully validate the RMIC-MT construct in Dutch for the elderly care setting, we were able to contribute to the evidence of its applicability and representativeness in the primary integrated care setting.

We found moderate rather than good results for a few of our fit indices. This could be the result of our relatively small sample size compared to the previous successful validation studies of the RMIC [16,17], as fit indices tend to indicate inappropriate fit in smaller sample sizes [29]. We found a slightly different factor structure than previous studies. This may be, because our explained variance for some of the domains may have differed from the previously validated RMIC-MT. As previous RMIC-MT validation studies already showed, the explained variance between studies varied for e.g. the domain cultural competence and care integration [16,17], which are likely due to cultural differences between countries or the difference in target condition (e.g. renal care or mental health care).

With this study, we add to the evidence that the RMIC-MT questionnaire is a promising tool to measure primary elderly care integration. Valid easy-to-use instruments to measure integrated care in the primary elderly care setting are lacking and available tools are of poor quality [10,11,12,13,14,30]. Almost all other available tools only focus on a single aspect of care integration, mainly person-focused care and clinical integration and do not represent normative and system integration [11]. The RMIC-MT questionnaire is based on the RMIC framework, and therefore covers all relevant domains of integrated care, therewith doing justice to complexity of integrated care [15,16,31].

We observed that thirteen percent of professionals did not complete the questionnaire. Reasons could be that the RMIC-MT is perceived as too time consuming or professionals find it difficult to interpret the questions. The last explanation is also supported by the fact that professionals frequently answered ‘I don’t know/neutral’. Since for system coordination, professional coordination, and technical competence the majority of the answers were ‘I don’t know/neutral’, we assume that the RMIC-MT questions for these topics might be outside the scope of the participating professionals. Extra explanatory notes might be necessary. Especially the answers given for system coordination can be explained, since professionals find it difficult to assess system domains of integrated care [4]. Perhaps system coordination policies are not experienced by professionals in daily practice and out of their scope of influence and therefore not recognizable. We did not expect and cannot explain why professionals had difficulties with answering questions about professional coordination as this is obviously part of their daily practice. Previous research shows that professionals focus and improve most on this domain [32] when developing their care integration. Possibly and despite pilot-testing, the terminology or formulation used in the questionnaire to describe this domain may not have matched the vocabulary of the participating professionals. This should be clarified in future research.

### Strengths and limitations

One of the strengths of this study is that we could build upon previous results from RMIC-MT construct validations for various conditions in primary care, which solidifies the results for this Dutch version for primary elderly care [16,17]. Moreover, this will contribute to the comparability of the results for different settings by using the same measurement tool.

Another strength is that we were able to include networks of elderly care professionals throughout the Netherlands, even though a large part of the networks were DementiaNet networks located in the east of the Netherlands. The participants also had varying professional backgrounds with all relevant disciplines represented.

A limitation is our sample size. We had a high number of non-responders, reasons for which remained unclear. However, data was collected during times of COVID-19, which potentially hampered the responses, since professionals were mainly focused on direct patient care. We reached 90% of the intended sample size (i.e. 323 of 360 participants). Although, literature suggests that 300 cases is generally sufficient for a CFA [33], our participants frequently did not finish the questionnaire which decrease power. For the CFI and TLI fit indices the results were considered moderate, for which the cause might have been the relatively small sample size. The results of the chi-square test tend to be less reliable with a small sample size and the other fit indices also tend to show a worse fit [29]. We recommend to perform a study with a larger sample size to identify whether these issues are then solved. Another limitation is the combined analysis of the ‘I don’t know’ option and the ‘neutral’ option: evidently these answers do not exactly mean the same. Since these two options both similarly indicate that the professional had no strong preference, this will most likely not affect our results. In future research, we suggest to remove the ‘I don’t know’ option.

### Implications for research and practice

Our CFA of the RMIC-MT for Dutch primary elderly care is a valuable first step for application of the RMIC-MT in this setting in the Netherlands. The RMIC-MT can be used for performance assessment (e.g. network evaluations) of the collaboration processes [9,16] in primary care networks. These assessments can reveal areas for further improvement of their care integration according to the different domains of integrated care, for example improving interprofessional communication by using a digital tool or by implementing a multidisciplinary meeting [9,16]. Moreover, insights from the RMIC-MT assessments can be used for educational purposes, thereby tailoring trainings to the needs of a network. In research, RMIC-MT has the potential to become the preferred instrument for measurement of care integration, as, with the current study included, it was already validated in different primary care settings and for various conditions [11].

Validation of the RMIC-MT for primary elderly care should be repeated in a larger sample size, to hopefully show good fit indices. Subsequently, future studies may include assessment of its feasibility and its applicability for improvement purposes. Eventually, application in intervention studies may show whether if the tool indeed enables networks to improve their care integration.

Although the vast majority of professionals filled in the entire questionnaire, we did receive incomplete questionnaires. A shorter and easier formulated version already showed positive results for physical and mental healthcare [34]. Moreover, identifying if the questions that are frequently answered with ‘I don’t know’ are not too difficult or irrelevant for this target group is essential. In this study, we focused on the RMIC-MT version for professionals. To give professionals an even more complete overview of their current care integration, incorporating multiple stakeholder perspectives’ is needed. It is therefore desirable to also include patients’ and informal caregivers’ experiences, by e.g. using the RMIC-MT patient version in addition to the professional version [16,35,36].

## Conclusion

We were able to build upon previous validations of the RMIC-MT and passed the majority of goodness-to-fit tests. This makes the RMIC-MT’s utility promising for the primary elderly care setting in the Netherlands, but not yet ready to assess integrated care in daily practice. Feasibility and added value of the tool should be studied after this small-scale validation study. The instrument has the potential to facilitate care integration, as it eventually could be used for evaluating integrated care initiatives in a primary care setting, thereby contributing to implementation of integrated primary elderly care.

## Additional File

The additional file for this article can be found as follows:

10.5334/ijic.6739.s1 Appendixes. Appendix I to Appendix V.
