# Supplementary material for: Construct Validation of the Rainbow Model of Integrated Care Measurement Tool in Dutch Primary Care for Older Adults
Source: Int J Integr Care. 2023 Feb 15;23(1):9. doi: 10.5334/ijic.6739 (PMC9936912; doi:10.5334/ijic.6739)
Supplement: Appendixes. — Appendix I to Appendix V. [file ijic-23-1-6739-s1.pdf]

APPENDIX I – Overview of the networks' geographical location in the Netherlands

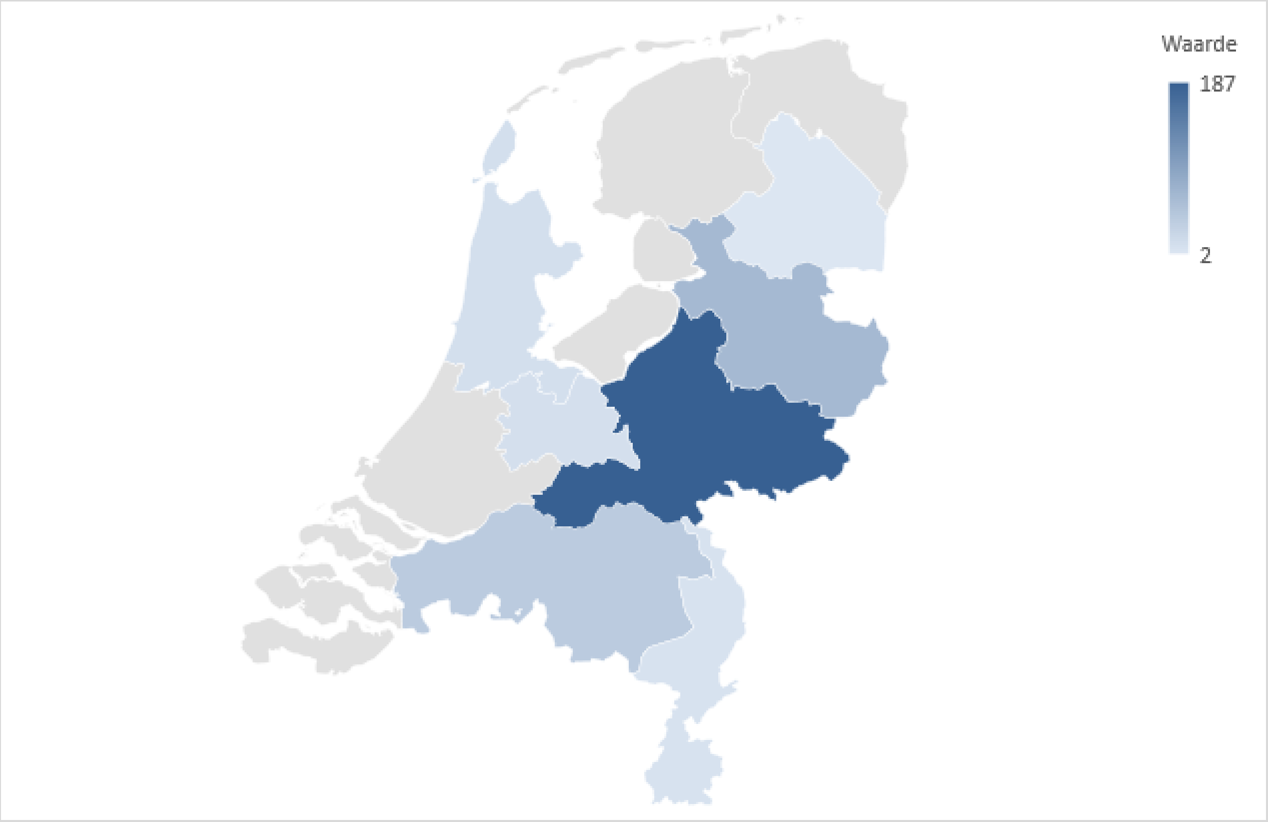

APPENDIX II – Rainbow Model of Integrated Care

The RMIC describes three categories of integrated care: the scope, type, and enablers of integration, including 8 domains. Figure 1 is a graphical representation of the RMIC.

Scope of integration

The scope entails the person-focused and population-focused view of professionals, e.g. focusing on patient's needs and abilities instead of the disease (person-focused care) and meeting a target group's specific healthcare requirements (population-focused care).

Type of integration

The type of integration consists of integration on the micro (individual), meso (population) and macro (system) level, and refers to four domains: 1) delivered and coordinated services to patients (clinical integration), 2) collaboration between healthcare professionals (professional integration), 3) collaboration between healthcare organisations (organisational integration) and 4) implementation of new policies and regulations (system integration).

Enablers of integration

Functional and normative enablers are needed to establish connectivity between the micro, meso and macro level. Functional enablers are for example communication tools that can be used by all professionals and organisations in a network, whereas normative enablers refer to the development and maintenance of a common goal or plans for improvement.

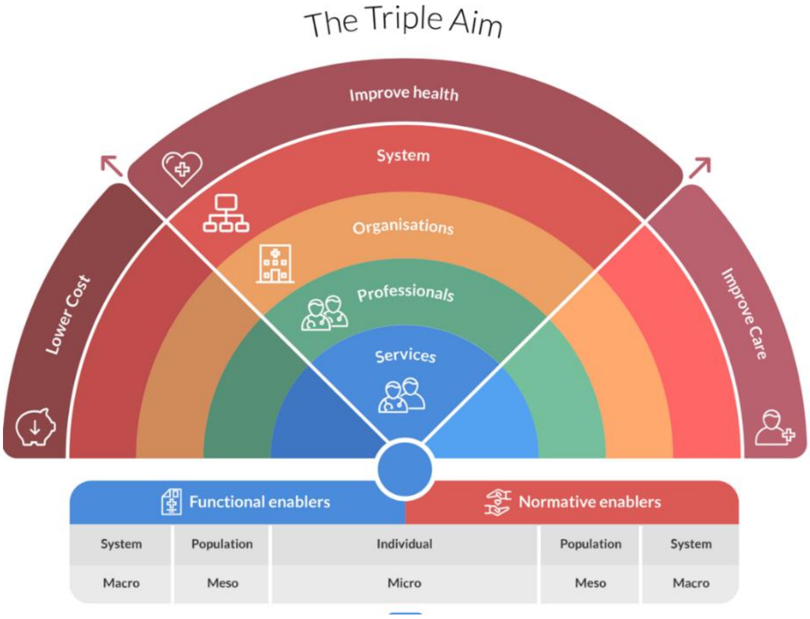

Figure 1. Rainbow Model of Integrated Care. Adapted with permission from Essenburgh Research & Consultancy [1].

Reference:

1. Essenburgh Research & Consultancy. *The Rainbow Model of Integrated Care*. 2017; Available from: https://www.essenburgh.com/en/rainbow-model-of-integrated-care.

APPENDIX III – Scale and response options of the 36-item RMIC-MT for primary elderly care professionals

Scale	Example	No. of items
Person-centred care	interventions are used to promote clients' self-care ability	5
Community-centred care	population needs are included in the objectives of the partnership	4
Clinical coordination	professionals have agreements on the referral and transfers (follow-up) of clients	3
Professional coordination	professionals use multidisciplinary guidelines and protocols	5
Organisational coordination	interest of the organisations involved are considered	3
System coordination	the partnership is hampered by the rules and/or policies set by the ministries (e.g. Ministry of health)	3
Technical competence	incentives are used to improve teamwork, coordination and continuity of care among professionals	4
Cultural competence	activities are undertaken to better understand other organisational cultures	4
Results-oriented	patient satisfaction is measured regularly	5

Based on Valentijn et al. (2015) [1]

Reference:

1. Valentijn PP, Vrijhoef HJ, Ruwaard D, Boesveld I, Arends RY, Bruijnzeels MA. Towards an international taxonomy of integrated primary care: a Delphi consensus approach. BMC Fam Pract. 2015;16: 64-015-0278-x.

APPENDIX IV – Pattern matrix of the Exploratory factor analysis for the RMIC-MT for primary elderly care professionals

	Component								
	1	2	3	4	5	6	7	8	9
**Safety.culture_8**	,788								
**Safety.culture_7**	,776								
**Safety.culture_5**	,726								
**Safety.culture_2**	,618								
**Safety.culture_6**	,617								
**Safety.culture_3**	,565								
**Safety.culture_1**	,493								
**External.care.coordination_3**		-,756							
**External.care.coordination_2**		-,748							
**External.care.coordination_7**		-,747							
**External.care.coordination_6**		-,731							
**External.care.coordination_1**		-,714							
**External.care.coordination_4**		,507		,349					
**External.care.coordination_5**		,364		,307					
**Internal.performance.management_4**			,768						
**Internal.performance.management_3**			,758						
**Internal.performance.management_2**			,679						
**Internal.performance.management_1**			,673						
**Internal.performance.management_5**			,638						
**Internal.performance.management_6**			,604						
**Internal.care.coordiation_6**				,718					
**Internal.care.coordiation_4**				,705					
**Internal.care.coordiation_7**				,601					
**Internal.care.coordiation_5**				,572					
**Internal.care.coordiation_2**				,569					
**Internal.care.coordiation_1**				,561					
**Internal.care.coordiation_3**				,534	,419				
**Person.centeredness_2**					,783				
**Person.centeredness_4**					,768				
**Person.centeredness_3**					,755				
**Person.centeredness_5**					,730				
**Person.centeredness_1**					,617				
**External.performance.management_2**						,755			
**External.performance.management_1**						,681			
**External.performance.management_4**						,644			
**External.performance.management_3**						,616			
**Laws.and.regulation_1**							,910		
**Laws.and.regulation_2**							,894		
**Laws.and.regulation_3**							,860		
**Community.centeredness_4**								,845	

**Community.centeredness_2**			,834	
**Community.centeredness_3**			,767	
**Community.centeredness_1**			,522	,312
**Regional.partnership_2**				,699
**Regional.partnership_3**	,330			,643
**Regional.partnership_4**				,566
**Regional.partnership_1**				,508
**Safety.culture_4**				,390

APPENDIX V – Correlation matrix of the RMIC-MT for primary elderly care professionals

	1	2	3	4	5	6	7	8	9	10	11	12	13	14	15	16	17	18	19	20	21	22	23	24	25	26	27	28	29	30	31	32	33	34	35	36
**1. Person.centeredness_1**	1																																			
**2. Person.centeredness_2**	,458**	1																																		
**3. Person.centeredness_3**	,482**	,474**	1																																	
**4. Person.centeredness_4**	,277**	,513**	,461**	1																																
**5. Person.centeredness_5**	,327**	,508**	,477**	,633**	1																															
**6. Community.centeredness_1**	,228**	,191**	,172**	,168**	,253**	1																														
**7. Community.centeredness_2**	,150**	,138*	0,107	,120*	,111*	,411**	1																													
**8. Community.centeredness_3**	,242**	,165**	,202**	,156**	,171**	,419**	,424**	1																												
**9. Community.centeredness_4**	,168**	,178**	,125*	,155**	,110*	,407**	,605**	,572**	1																											
**10. Internal.care.coordination_1**	0,103	0,106	,186**	,121*	,173**	,143*	,168**	,119*	,209**	1																										
**11. Internal.care.coordination_2**	0,106	0,102	,162**	,157**	,198**	,231**	,225**	,149**	,222**	,533**	1																									
**12. Internal.care.coordination_3**	,261**	,292**	,346**	,377**	,326**	,218**	,121*	,146*	,119*	,238**	,380**	1																								
**13. External.care.coordination_1**	-,172**	-0,094	-,170**	-,161**	-,123*	-0,103	-0,085	-0,097	-,147*	-,154**	-,241**	-,142*	1																							
**14. External.care.coordination_2**	-,125*	-0,029	-,117*	-0,075	-0,095	-0,093	0,000	-0,070	-0,059	-,134*	-,122*	-0,107	,556**	1																						

**15. External.care.coordination_3**	-0,107	-0,039	-,157**	-0,070	-0,091	-,140*	-0,093	-0,078	-0,092	-,118*	-0,077	-0,047	,475**	,426**	1																				
**16. External.care.coordination_6**	-0,087	-0,036	-,129*	-0,018	-0,081	-0,102	-0,030	-0,083	-0,005	-,136*	-0,094	-0,108	,421**	,405**	,389**	1																			
**17. External.care.coordination_7**	-,135*	-0,054	-0,113	0,001	-0,026	-,141*	-,119*	-0,068	-,142*	-,183**	-,175**	-,126*	,453**	,461**	,425**	,479**	1																		
**18. Regional.partnership_2**	,152**	0,103	0,000	0,107	0,069	,213**	,147*	0,071	,202**	,160**	,152**	0,080	-,162**	-0,076	-0,054	-0,062	-,123*	1																	
**19. Regional.partnership_3**	0,065	0,045	0,024	0,078	0,064	,206**	,161**	,126*	,242**	,116*	,196**	,150**	-,188**	-0,034	-,154**	-0,110	-,141*	,395**	1																
**20. Regional.partnership_4**	0,112	0,080	0,066	0,099	0,079	,278**	,224**	,187**	,321**	,221**	,203**	,200**	-,188**	-0,032	-0,101	-0,045	-,134*	,412**	,449**	1															
**21. Laws.and.regulation_1**	0,064	-0,052	0,046	0,024	0,036	0,023	,150**	0,076	,138*	,114*	0,083	0,094	-0,093	-0,011	-0,099	-0,097	-0,046	,130*	0,084	,142*	1														
**22. Laws.and.regulation_2**	0,043	0,003	0,034	-0,035	0,057	0,095	,164**	0,059	,121*	,142*	,119*	0,075	-0,081	-0,006	-0,060	-,139*	-0,056	,172**	0,087	,198**	,732**	1													
**23. Laws.and.regulation_3**	0,042	-0,002	0,041	0,010	0,068	,125*	0,108	0,086	,130*	,180**	,125*	0,060	-,114*	-0,069	-,153**	-,182**	-0,040	,166**	,149*	,211**	,697**	,723**	1												
**24. Internal.performance.management_1**	,152**	,120*	,183**	,197**	,252**	,224**	,127*	,182**	,220**	,373**	,350**	,200**	-,207**	-,201**	-0,080	-0,114	-,179**	,177**	,226**	,236**	,152**	0,111	,233**	1											
**25. Internal.performance.management_2**	,152**	,203**	,126*	,158**	,176**	0,107	,236**	,223**	,269**	,335**	,280**	,242**	-0,114	-0,113	-0,039	0,037	-0,072	,201**	0,107	,170**	,225**	,224**	,272**	,462**	1										
**26. Internal.performance.management_3**	,175**	,151**	,123*	,174**	,274**	,151**	0,099	,167**	,167**	,336**	,332**	,263**	-,197**	-,172**	-0,069	-,141*	-0,099	,219**	,231**	,169**	,168**	,185**	,240**	,551**	,527**	1									
**27. Internal.performance.management_4**	,170**	,191**	,198**	,173**	,268**	,215**	0,094	,192**	,186**	,333**	,379**	,199**	-,225**	-,172**	-0,106	-,117*	-,171**	,211**	,227**	,230**	0,070	,160**	,173**	,543**	,431**	,653**	1								
**28. Internal.performance.management_5**	0,055	,119*	0,093	,159**	,178**	0,057	,195**	0,063	,208**	,223**	,241**	0,067	-0,105	-0,104	-0,022	-0,004	-0,070	,175**	,152**	,222**	,262**	,296**	,233**	,400**	,411**	,441**	,424**	1							
**29. External.performan**	0,073	0,027	0,008	-0,0	-0,0	0,024	0,010	,136*	0,098	,228**	,167**	0,063	-,15	-0,0	-,14	-0,0	-0,0	,225**	,387**	0,115	0,090	,125*	,183**	,167**	,193**	,183**	,231**	,203**	1						

**ce.management_1**				17	47								2**	52	6*	42	57																			
**30. External.performance.management_2**	-0,004	0,013	-0,102	-0,042	-0,076	0,052	0,080	0,072	,120*	,224**	,135*	0,018	-0,108	-0,056	-,127*	-0,026	-0,045	,255**	,306**	,172**	0,077	,131*	,161**	,156**	,196**	,199**	,175**	,179**	,629**	1						
**31. External.performance.management_3**	,129*	0,040	0,114	-0,031	0,025	,133*	,152**	0,108	,191**	,266**	,245**	,123*	-,130*	-0,096	-,190**	-0,070	-,150*	,226**	,228**	,298**	0,091	0,111	,147*	,241**	,193**	,231**	,202**	,158**	,301**	,398**	1					
**32. External.performance.management_4**	0,038	0,073	0,031	0,030	0,042	0,026	,161**	0,026	,152**	,254**	,271**	0,097	-0,050	0,015	-,140*	-0,036	-0,069	,136*	,154**	,286**	0,111	,139*	,132*	,209**	,233**	,231**	,189**	,390**	,277**	,388**	,580**	1				
**33. Safety.culture_1**	,284**	,264**	,191**	,128*	,189**	,323**	,139*	0,089	,141*	,220**	,279**	,206**	-,164**	-,160**	-,147*	-0,054	-0,057	,218**	,144*	,207**	0,092	,136*	,120*	,229**	,193**	,243**	,239**	,157**	0,115	0,022	,150*	0,084	1			
**34. Safety.culture_3**	,207**	,189**	,178**	,123*	,189**	,321**	,158**	,201**	,202**	,260**	,301**	,192**	-,161**	-0,043	-,131*	-,122*	-,124*	,259**	,263**	,276**	,135*	,175**	,169**	,254**	,232**	,309**	,430**	,190**	,159**	,118*	,239**	,160**	,435**	1		
**35. Safety.culture_4**	0,115	,119*	0,047	0,027	0,108	,167**	0,026	0,071	,118*	0,072	,171**	0,044	-,142*	-0,082	-0,084	-0,006	-0,076	,152*	,124*	,148*	-0,048	0,095	0,013	,120*	0,097	0,094	,176**	0,085	0,040	0,025	0,042	0,030	,417**	,228**	1	
**36. Safety.culture_5**	,158**	,128*	0,085	0,093	,122*	,131*	0,049	0,025	0,066	,285**	,197**	,125*	-,186**	-0,071	-,161**	-,156**	-0,072	,218**	,230**	,228**	0,058	0,067	,156**	,147*	,268**	,217**	,149*	,180**	,187**	,184**	,259**	,232**	,375**	,505**	,148*	1

*P<0.05
**P<0.01
